# Elevated estradiol levels in frozen embryo transfer have different effects on pregnancy outcomes depending on the stage of transferred embryos

**DOI:** 10.1038/s41598-022-09545-7

**Published:** 2022-04-04

**Authors:** Qing Li, Liming Ruan, Lingling Zhu, Zengyu Yang, Maoling Zhu, Yudi Luo

**Affiliations:** 1 Reproductive Medicine Center, Nanning Maternity and Child Health Hospital, Nanning, 530011 China; 2 Reproductive Medicine Center, Yulin Maternity and Child Health Hospital, Yulin, 537000 China

**Keywords:** Endocrinology, Medical research

## Abstract

Supplementation with estradiol (E_2_) is routinely used in frozen embryo transfer (FET) cycles and embryo age plays an important role in conceiving. This study was to compare the effects of serum E_2_ levels on pregnancy outcomes between cleavage- and blastocyst-stage FET cycles using hormone replacement therapy. A total of 776 FET cycles (669 couples) performed from January 2016 to December 2019 were included in the present retrospective cohort study. Regarding cleavage-stage embryo transfers, E_2_ levels on progesterone initiation day were significantly lower in the ongoing pregnancy/live birth (OP/LB) group than in the non-OP/LB group (214.75 ± 173.47 vs. 253.20 ± 203.30 pg/ml; *P* = 0.023). In addition, there were downward trends in implantation, clinical pregnancy and OP/LB rates with increasing E_2_ levels. However, in blastocyst-stage embryo transfers, such trends were not observed, and E_2_ levels were not significant difference between the OP/LB group and the non-OP/LB group (201.66 ± 182.14 vs. 197.89 ± 212.83 pg/ml; *P* = 0.884). The results suggests that elevated progesterone-initiation-day E_2_ levels may negatively affect pregnancy outcomes during artificial cleavage-stage embryo transfers. However, it is not necessary to monitor E_2_ levels when transferring blastocysts in artificial FET cycles.

## Introduction

Endometrial preparation is a critical step in both the natural and artificial frozen embryo transfer (FET) cycle because the development of the endometrium must be synchronized with the embryo transfer for successful implantation^1^. Undoubtedly, estradiol (E_2_) coordinately interacts with progesterone and plays an important role in endometrial development^2,3^. In fresh cycles, excess E_2_ has been shown to be detrimental to endometrial development and to ultimately adversely affect conception^4,5^. Moreover, excess E_2_ levels have been found to increase the incidence of abnormal pregnancy conditions, such as intrauterine growth restriction and abnormal implantation of the placenta^6,7^. There is limited available information regarding the need for endocrine monitoring during hormone replacement therapy (HRT), although supplementation with steroid hormones is necessary for endometrial preparation. Fritz et al. demonstrated that elevated E_2_ levels in artificial FET cycles were related to a low ongoing pregnancy/live birth (OP/LB) rate^8^, but some authors did not observe this association^9–11^. The discrepant findings may be due to the different patient populations recruited, differences in the protocols used for endometrial preparation, and differences in the day in which steroid hormone levels were measured between studies. In addition to synchronization between endometrial maturation and transferred embryos, female age, the uterine condition, and embryonic factors are known as major variables affecting embryo implantation. To eliminate bias due to the effects of other variables on embryonic implantation, the present retrospective study analyzed frozen-thawed transfer cycles with two good-quality embryos transferred into the normal uterus of patients under 40 years of age with an endometrial thickness of at least 7 mm on the progesterone initiation day. Cleavage-stage embryo transfer cycles and blastocyst-stage embryo transfer cycles were analyzed separately and then compared in this study because the age of the transferred embryo is an important factor affecting the success rate of implantation.

The aim of this study is to compare the effects of serum E_2_ on pregnancy outcomes between cleavage- and blastocyst-stage FET cycles using HRT. To our knowledge, to date, this is the largest study examining E_2_ levels on progesterone initiation day on the effects of OP/LB between cleavage- and blastocyst-stage FET cycles.

## Results

A total of 776 FET cycles (669 patients) were retrospectively reviewed from January 2016 to December 2019. The implantation rate, clinical pregnancy rate (CPR) and OP/BL rate were 27.4%, 43.3% and 33.5%, respectively, for cleavage-stage embryo transfer cycles and 48.1%, 69.3%, 59.2%, respectively, for blastocyst-stage embryo transfer cycles.

As shown in Table 1, there were no significant differences between OP/BL and non-OP/BL for cleavage-stage embryo transfer cycles regarding body mass index, type of infertility, basal serum follicle-stimulating hormone levels on cycle day 2–3, transferred embryo quality, serum levels of progesterone and luteinizing hormone, or endometrial thickness on progesterone initiation day. However, the average female age, days of E_2_ administration, serum E_2_ level on progesterone initiation day, and duration of infertility were significantly lower in the OP/BL group than in the non-OP/BL group (31.9 vs. 33.4 years, *P* < 0.001; 15.30 vs. 16.14 days, *P* = 0.002; 214.75 vs. 253.20 pg/ml, *P* = 0.023; 4.3 vs. 5.0 years, *P* = 0.009; respectively). For blastocyst-stage embryo transfer cycles, no significant differences in any of the variables were observed between the OP/BL and non-OP/BL groups.

**Table 1:** Demographic details between OP/LB women and non-OP/LB women.

Parameters	Cleavage-stage embryos			Blastocyst-stage embryos		
	OP/LB (n = 180)	Non-OP/LB (n = 358)	*P*-value	OP/LB (n = 141)	Non- OP/LB (n = 97)	*P*-value
Female age (years)	31.94 ± 3.84	33.39 ± 3.82	< 0.001	31.56 ± 4.27	31.84 ± 4.25	0.626
BMI (kg/m^2^)	22.10 ± 2.74	21.97 ± 2.82	0.611	22.02 ± 3.06	22.22 ± 3.11	0.618
Basal FSH (mIU/ml)	5.94 ± 2.29	6.21 ± 2.62	0.236	5.47 ± 1.81	5.59 ± 2.33	0.636
**Type of infertility**			0.802			0.458
Primary	82 (34.0)	159 (66.0)		60 (56.6)	46 (43.4)	
Secondary	98 (33.0)	199 (67.0)		81 (61.4)	51 (38.6)	
Duration of infertility (years)	4.3 ± 2.9	5.0 ± 3.7	0.009	4.1 ± 2.9	3.9 ± 2.4	0.432
**Transferred embryo quality**			0.363			0.295
1	117 (35.8)	210 (64.2)		54 (60.0)	36 (40.0)	
2	37 (30.1)	86 (69.9)		43 (53.1)	38 (46.9)	
3	26 (29.5)	62 (70.5)		44 (65.7)	23 (34.3)	
E_2_ administration (days)	15.30 ± 2.50	16.14 ± 3.36	0.002	16.15 ± 3.27	16.26 ± 3.13	0.798
**E**_**2**_** on progesterone initiation day (pg/ml)**						
	214.75 ± 173.47	253.20 ± 203.30	0.023	201.66 ± 182.14	197.89 ± 212.83	0.884
**Progesterone on progesterone initiation day (ng/ml)**						
	0.35 ± 1.51	0.27 ± 0.74	0.427	0.20 ± 0.16	0.21 ± 0.17	0.584
**LH on progesterone initiation day (mIU/ml)**						
	8.00 ± 9.16	7.21 ± 7.96	0.304	7.10 ± 7.84	7.56 ± 8.95	0.674
**Endometrial thickness on progesterone initiation day (mm)**						
	9.23 ± 1.21	9.11 ± 1.22	0.251	8.95 ± 1.29	9.04 ± 1.37	0.630

Data are presented as mean ± standard deviation or n (%).

*OP/LB* ongoing pregnancy/live birth, *BMI* body mass index, *FSH* follicle stimulating hormone, *E*_*2*_ estradiol, *LH* luteal hormone.

To further investigate serum E_2_ levels on the effect of pregnancy outcomes, patients were classified into three percentile groups based on the serum E_2_ level on progesterone initiation day: the 1st–10th percentile (group 1), the 11th–90th percentile (group 2) and the 91st–100th percentile (group 3). As shown in Table 2, we observed decreasing trends in the implantation rate (*P* < 0.001), CPR (*P* = 0.001) and OP/BL rate (*P* = 0.024) with increasing E_2_ levels for cleavage-stage embryo transfer cycles. Cycles in the group 3 (E_2_ range: 508.4–951.0 pg/ml) had a significantly lower OP/BL rate compared to cycles in the group 1 (E_2_ range: 2.7–47.1 pg/ml) (18.9% vs. 43.4%, *P* = 0.006) and in the group 2 (E_2_ range: 47.4–504.6 pg/ml) (18.9% vs. 34.0%, *P* = 0.026). However, such trends were not observed for the implantation rate, CPR or OP/BL rate in blastocyst-stage embryo transfer FET cycles.

**Table 2:** Pregnancy outcomes according to percentile analysis of estradiol levels. Data are presented as n (%).

	Cleavage-stage embryos				Blastocyst-stage embryos			
	Group 1 (n = 53)	Group 2 (n = 432)	Group 3 (n = 53)	*P*-value	Group 1 (n = 23)	Group 2 (n = 192)	Group 3 (n = 23)	*P*-value
Implanting rate	39 (36.8)	242 (28.0)	14 (13.2)	< 0.001	29 (63.0)	179 (46.6)	21 (45.7)	0.102
Clinical regnancy rate	30 (56.6)	191 (44.2)	12 (22.6)	0.001	19 (82.6)	131 (68.2)	15 (65.2)	0.333
Ongoing pregnancy/live birth rate	23 (43.4)	147 (34.0)	10 (18.9)	0.024	16 (69.6)	113 (58.9)	12 (52.2)	0.472
Miscarriage rate	7 (23.3)	44 (23.0)	2 (16.7)	0.875	3 (15.8)	18 (13.7)	3 (20.0)	0.798

Generalized estimating equation was performed to assess the correlations between the OP/BL rate and the variables that the *P* value was under 0.10, as shown in Table 1. These variables included female age, duration of infertility, days of E_2_ administration, and serum E_2_ on progesterone initiation day for cleavage-stage embryo transfer cycles. The results revealed that female age (odds ratio (OR) 0.906, 95% confidence interval (95% CI) 0.858–0.956, *P* < 0.001) and days of E_2_ administration (OR 0.912, 95% CI 0.854–0.973, *P* = 0.006) were independently associated with the OP/BL rate for cleavage-stage embryo transfer cycles (Table 3). Generalized estimating equation for blastocyst-stage embryo transfer cycles was not performed because no *P* value was under 0.10, as shown in Table 1.

**Table 3:** Valuables associated with OP/BL analyzed by generalized estimating equation for cleavage-stage embryo transfer.

Valuables	OR	95% CI	*P* value^a^
Female age	0.906	0.858–0.956	< 0.001
Duration of infertility	0.966	0.908–1.028	0.278
Days of E_2_ administration	0.912	0.854–0.973	0.006
E_2_ level on progesterone initiation day	1.000	1.000–1.000	0.176
Constant	75.021	8.997–625.591	< 0.001

*OP/LB* ongoing pregnancy/live birth, *E*_*2*_ estradiol, *OR* odds ratio, *CI* confidence interval.

^a^After controlling for confounders (female age, duration of infertility, days of E_2_ administration, and serum E_2_ levels on progesterone initiation day).

**Figure 1:**
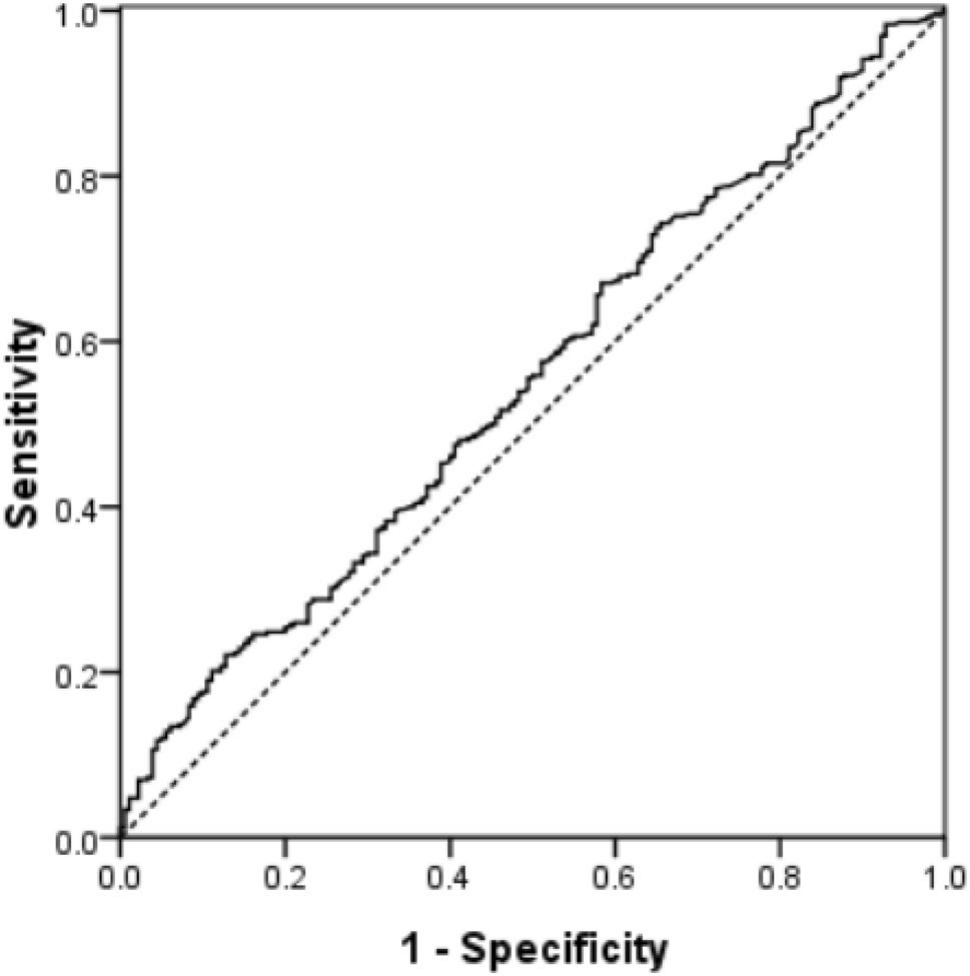
Receiver operator characteristic curve for prediction of ongoing pregnancy/live birth for cleavage-stage embryo transfers. The area under the curve is 0.55.

Since elevated E_2_ level was associated with low pregnancy rates in cleavage-stage embryo transfer cycles, receiver operating characteristic (ROC) analysis was generated to determined whether the serum E_2_ level on progesterone initiation day could predict OP/BL in these cycles. The result revealed that E_2_ level of ≥ 413.6 pg/ml was associated with low chance of OP/LB, the positive predictive value for these was 22.1%, while the negative predictive value was 87.2% and the areas under the ROC curve were 0.55 (95% CI 0.50–0.60) (Fig. 1).

## Discussion

We demonstrated that the serum E_2_ level on the progesterone initiation day was significantly higher in the non-OP/LB group than in the OP/LB group during artificial FET cycles with cleavage-stage embryo transfers. In addition, elevated E_2_ levels in the 91st–100th percentile had detrimental effects on embryo implantation, clinical pregnancy and OP/LB compared to E_2_ levels in the 1st–10th percentile and in the 11th–90th percentile. However, such effects of E_2_ levels on embryo implantation, clinical pregnancy and OP/LB were not observed in frozen-thawed blastocyst-stage embryo transfer cycles. This is the first study to find that serum E_2_ levels on progesterone initiation day have different effects on pregnancy outcomes between cleavage-stage embryo transfers and blastocyst-stage embryo transfers during artificial FET cycles.

Although estrogen levels in normal natural cycles reach 300–400 pg/ml before ovulation, a study on donor cycles revealed that the E_2_ requirement for embryo implantation is low (< 100 pg/ml)^12^. Our study supports this conclusion, showing approximately 40% of conceptions occurring in patients with E_2_ levels < 100 pg/ml either in cleavage-stage embryo transfers (95/233) or in blastocyst-stage embryo transfers (65/165). On the other hand, successful conception in fresh embryo transfer cycles implies that embryo implantation can occur in an environment with the supraphysiological levels of E_2_ caused by ovarian stimulation. Our data showed that pregnancies could occur in a wide range of serum E_2_ levels from low levels of 10 pg/ml to a supraphysiological level of > 1000 pg/ml.

It has been suggested that blastocyst stage transfer is associated with higher rates of clinical pregnancies than cleavage stage transfer after Glujovsky et al. reviewed 27 randomised controlled trials^13^. Therefore we analyzed separately cleavage-stage and blastocyst-stage embryo transfers in order to diminish the bias caused by this factor. Interestingly, we found that the impact of E_2_ on pregnancies in these two groups were different. Regarding cleavage-stage embryo transfers, elevated E_2_ levels in the 91st–100th percentile on progesterone initiation day had detrimental effects on CPR and OP/LB rates in HRT cycles compared to the lower E_2_ levels in the 1st–10th percentile and in the 11th–90th percentile. Fritz et al. had a similar finding that OP/LB decreased six-fold in FET cycles with the highest 10% E_2_ concentrations compared to those cycles with the lowest 10% E_2_ concentrations^8^. The detrimental effects of an elevated E_2_ level on pregnancy have been reported in fresh in vitro fertilization cycles^4,5,14^. Experiments with a mouse model revealed that high levels of estrogen rapidly close the receptivity window for embryo implantation by altering endometrial gene expression, which causes the uterus to become unreceptive for implantation^15,16^. As a consequence of this shortened receptivity window caused by a high E_2_ level, the receptivity window may be missed for a late-growing blastocyst (equivalent to day 6 or day 7 blastocysts) that developed from a cleavage-stage embryo transfer, resulting in a low chance of implantation. In our lab, 37.9% viable embryos observed on day 3 would develop finally to blastocysts on day 5 or day 6. Considering day 6 blastocysts would miss the receptivity window, we do not transfer day 6 blastocysts in fresh cycles. However, a blastocyst transferred in HRT cycles can result in immediate implantation within the receptivity window even if the receptivity window is shorten by elevated E_2_ level. This can be explained in our study that serum E_2_ levels were not associated with pregnancy outcomes in blastocyst transfer cycles. Moreover, transferring with better quality of blastocysts resulted in higher pregnancy rates compared to cleavage-stage embryo transfers, which may have diminished the detrimental effect of elevated E_2_ on pregnancy outcomes for the blastocyst transfers. Similarly, in a study by Özdemir et al., E_2_ levels did not have significant effects on the pregnancy and miscarriage rate in autologous day 5 embryo transfer cycles using HRT^10^.

In contrast to our findings regarding high E_2_ levels in cleavage-stage FET cycles, some authors have not found an adverse effect of high E_2_ levels on pregnancy outcomes. Niu et al. reported that E_2_ concentrations did not appear to be associated with the pregnancy rate in autologous day-3 FET cycles^9^. They grouped E_2_ concentrations on progesterone initiation day into the 0–25th, 25th–75th, and 75th–100th percentile groups. The average E_2_ concentration in the highest group (299 ± 48.9 pg/ml) in their study was not in the range of the highest 10th percentile of our study (508.4–951.0 pg/ml). When we regrouped E_2_ levels according to their study, no differences in pregnancy or implantation between groups were observed. Classifying E_2_ levels into five groups, Remohi et al. did not find elevated E_2_ levels on the oocyte donation day to be associated with the implantation or pregnancy rate^12^. This may be due to the high quality of embryos derived from donor oocytes that can be implanted within a narrow receptive window in conditions of elevated E_2_ levels, which was similar to our findings for blastocyst-stage embryo transfer cycles. Mackens et al. did not observed serum E_2_ levels had significant difference within three groups classified by percentile similar to ours in terms of LBR after FET^17^. In this study, transferred embryos including cleavage-stage and blastocyst-stage were analysed together which may account for the discrepant findings. In contrast to our finding of a significant difference in E_2_ levels between the OP/LB group and the non-OP/LB group for cleavage-stage embryo transfer cycles, this difference in E_2_ levels was not found by several authors^8,11,18^. This discrepancy maybe have two main causes. First, these authors used transdermal and intramuscular E_2_ for E_2_ supplementation, whereas we used oral estrogen. The oral route of adminstration with E_2_ valerate is metabolized by 17β-hydroxysteroid dehydrogenase into estrone (E_1_) after absorption in the small intestinal mucosa and first pass through the liver. E_1_ has a weaker estrogenic activity with lower binding affinity for both α and β receptors compared with E_2_^19,20^. On the results of these transitions, approximately normal serum E_2_ levels can be obtained by oral route. When estrogen is administered transdermally, intramuscularly or vaginally, without the first-pass metabolism of E_2_ in the liver, E_2_/E_1_ ratios are higher than in oral administration. Therefore an E_2_ patch with 0.1 mg dose produces similar bioactivity to an oral dose of 2 mg^20^. However, the oral route of administration is the first option in our clinic to prepare endometrium for embryo implantation since it is easy to use and relatively inexpensive. Second, the study by Fritz et al. pooled day-3 and day-5 embryos for analysis^8^, whereas we analyzed data for cleavage-stage embryo transfers and blastocyst-stage embryo transfers separately.

Generalized estimating equation was conducted to analyze E_2_ levels on progesterone initiation day in cleavage-stage embryo transfer cycles. We found that both the days of E_2_ administration and female age were independent variables affecting OP/LB. This results implied that days of E_2_ administration significantly affected OP/LB for cleavage-stage embryo transfers through serum E_2_ in oral route administration. Interestingly, Mackens et al. found embryo quality was the only parameter with significant impact in the regression model for the best embryo transferred^17^. There is no doubt that embryo quality is important for successful embryo implantation. In our study, higher success rates in OP/LB receiving by two good embryo transfer may hide the importance of embryo quality. ROC curve analysis revealed that the E_2_ level of ≥ 413.6 pg/ml on progesterone initiation day was associated with low OP/LB rate in these cycles. Therefore when transferring cleavage-stage embryos in FET cycles, avoiding E_2_ levels exceeding a threshold 413.6 pg/ml by adjusting E_2_ dosage may be benefit for pregnancy outcomes. But E_2_ levels can not predict OP/LB.

We noticed that there were high variation in the E_2_ levels ranged from 2.72 to 1142.78 pg/ml. It has been reported that E_2_ reached a maximum at 5 h, remained significantly elevated at 8 h, and dropped down to baseline levels at 24 h after oral administration containing 2 mg micronized E_2_^21^. In our clinic, patients in HRT cycles were requested to take E_2_ valerate orally twice a day for endometrial preparation, one time in the morning and another time 12 h later, in order to keep serum E_2_ in a relative stable levels. Blood samples for serum E_2_ measurements were drawn at 8–9 O’clock in the morning before E_2_ administration with the purpose to diminish variation of E_2_ levels. However, one of the limitation of the study is that we could not fix the time interval between the last oral intake of E_2_ valerate and the collecting of the blood sample for serum E_2_ measurements, which could have led to variation of E_2_ levels. Meanwhile, due to the limitation of its retrospective nature in this study, we could collect only a single E_2_ measurement which may not be enough for interpretation of E_2_ value. Although E_2_ was administrated in one way by oral route, which dininished the variation caused by different routes of E_2_ administration, different progesterone preparation used in this study may also affect the clinic outcomes. Another limitation is the small sizes of the highest and lowest E_2_ levels. All the above limitations indicates more data is needed in a prospective study for assessing clinical value of serum E_2_ levels. The strength of this study is the recruitment of women under 40 years old with two good embryos being transferred into a normal endometrium and then evaluating the effects of E_2_ in cleavage-stage transfers and in blastocyst-stage transfers individually which eliminated the bias caused by other confounding variables.

In conclusion, our data indicate that excess serum E_2_ levels on the progesterone initiation day have detrimental effects on embryo implantation, pregnancy and OP/LB rates when cleavage-stage frozen embryos are transferred into the endometrium prepared by oral administration of E_2_ but have no adverse effect on frozen blastocyst-stage embryo transfers. Therefore, it is not necessary to monitor E_2_ levels in blastocyst-stage embryo transfer cycles. More data is needed to confirm the serum E_2_ value in clinical practice when transferring frozen cleavage-stage embryos.

## Methods

### Patients

**Figure 2:**
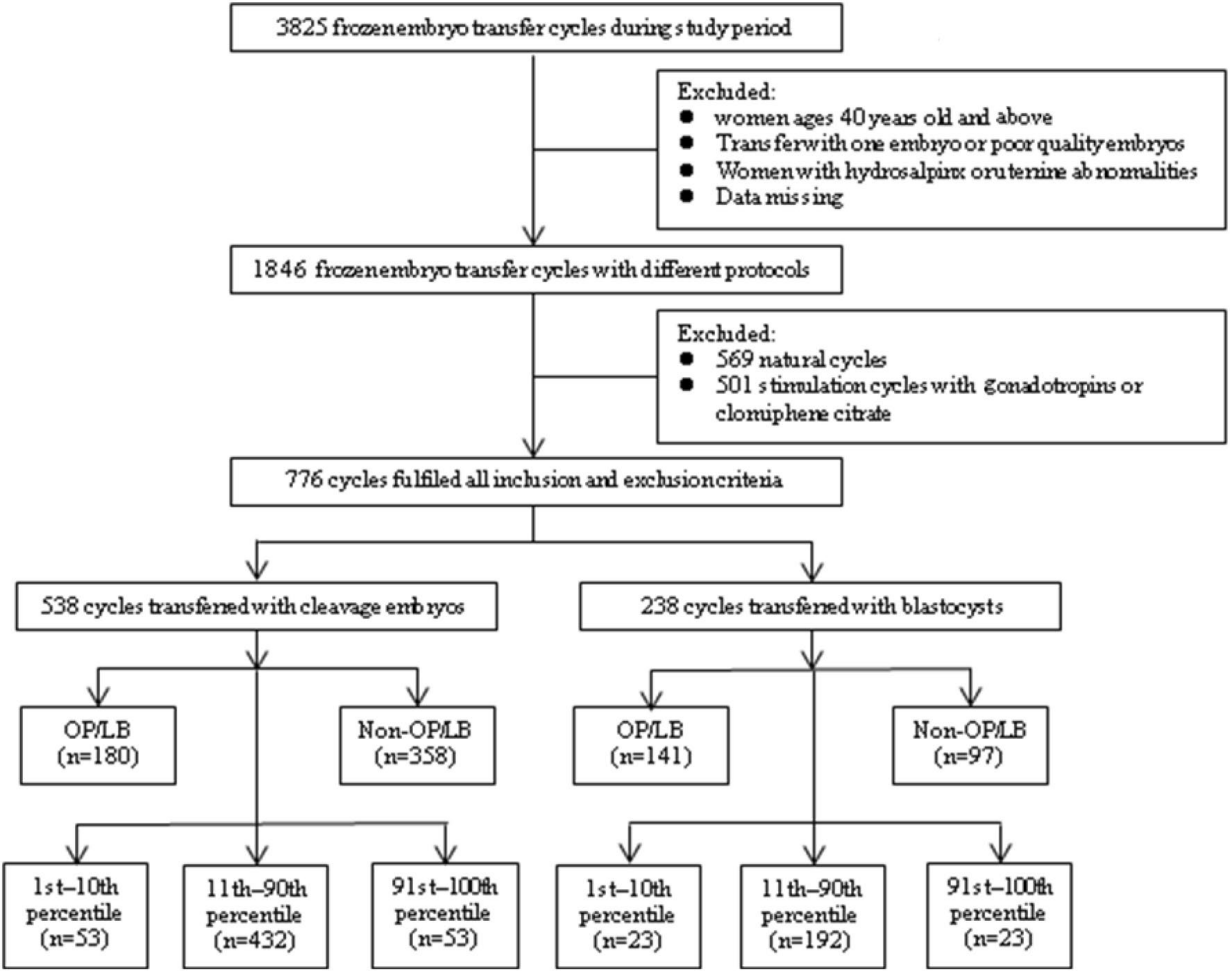
Flowchart of study design.

All FET cycles performed from January 2016 to December 2019 were retrospectively analyzed in this study. Basically, HRT cycles were provided in those anovulatory patients for endometrial preparation whereas natural cycles were the first options in ovulatory patients in our clinic. However, some patients who failed in previous FET cycles or other reasons may used HRT cycles. There was 3825 FET cycles during this period. A total of 776 HRT cycles (669 couples) that met the following criteria were included: women under 40 years of age, transfer of two viable-quality embryos, and endometrial thickness ≥ 7 mm on the progesterone initiation day (Fig. 2). Those day-3 embryos with at least 6 blastomeres and less than 40% fragmentation were defined as viable embryos. Among them, those with at least 7 even and homogeneous blastomeres and less than 20% fragmentation were considered as top quality embryos^22^. Blastocysts were assessed on day 5 or day 6 after oocyte collection according to Gardner’s criteria^23^, and all blastocysts except for CC (poor inner cell mass and poor trophectoderm) were defined as viable embryos. The two transferred embryos were scored as 1–3 according to their quality (Supplementary Table 1). Cryopreservation and thawing of embryos were performed with the Cryotop protocol reported by Kuwayama et al.^24^. Patients with hydrosalpinx or uterine abnormalities such as fibroids or congenital structural anomalies that may affect implantation were excluded. The present study was approved by Medical Ethics Committee of Yulin Maternity and Child Health Hospital (study number SZ20200319-1). The need for informed consent was waived by the above ethics committee owing to its retrospective nature. All methods were conducted in accordance with the relevant guidelines and regulations.

### Endometrial preparation and embryo transfer

Endometrial preparation started on days 2 to 3 of the cycle with a step-up dose of oral E_2_ valerate (Progynova, Bayer) with or without downregulation by gonadotropin-releasing hormone agonist. In general, 4 mg per day of E_2_ valerate was administered for the first 4 days, and then increased to 6 mg per day for the second 4 days, followed by the measurement of endometrial thickness by vaginal ultrasound. If endometrial thickness reached 7 mm, administration of the same dose of valerate was continued for 4 more days, and if not, the doses were increased to 8 mg per day onwards. When the thickness reached 7 mm after 12–15 days of E_2_ valerate administration, progesterone supplementation was administered by three routes based on patient preference: oral administration of 10 mg twice a day, 90 mg vaginal administration daily or 40 mg intramuscular injection daily. All patients underwent transfers of 2 embryos under ultrasound guidance with day-3 embryos 4 days after progesterone administration or blastocysts 6 days after progesterone administration. Serum levels of E_2_, progesterone and luteinizing hormone were measured on the progesterone initiation day using electrochemiluminescent immunoassay (Roche, Cobase, Switzerland). Human chorionic gonadotropin was examined to evaluate pregnancy on days 12–14 after embryo transfer. When pregnancy was achieved, the combination of E_2_ valerate and progesterone supplementation continued until 10–12 weeks of gestation.

### Pregnancy outcomes

The presence of a gestational sac and fetal heartbeat was considered a clinical pregnancy. The implantation rate was calculated as the number of gestational sacs by ultrasound observation divided by the number of transferred embryos. Live birth was defined as delivery of a viable infant after 24 weeks gestation. Miscarriage was diagnosed as spontaneous pregnancy loss.

### Statistical analysis

Statistical analysis was conducted with SPSS version 17 (SPSS Inc., Chicago, IL, USA). Continuous values are presented as the mean ± SD. Categorical values are expressed as percentages (%). Normality was assessed with the Shapiro–Wilk test. Student’s t-test or Mann–Whitney U test were used to evaluate differences between continuous variables based on the distribution. Categorical values were analyzed with the chi-square test. Since there were repeated patients in the data, generalized estimating equation with unstructured was performed to assess the correlations between the variables and the OP/LB rate. All the variables reaching a significance level of 0.10 were entered into the equation model. To determine the predictive power of E_2_ level on the effect of OP/LB, ROC curve was generated to achieve the cutoff and the area under the curve. A two-tailed *P* value < 0.05 was considered statistically significant.

## Supplementary Information

Supplementary Table 1.
