# Supplementary material for: Elevated estradiol levels in frozen embryo transfer have different effects on pregnancy outcomes depending on the stage of transferred embryos
Source: Sci Rep. 2022 Apr 4;12:5592. doi: 10.1038/s41598-022-09545-7 (PMC8980097; doi:10.1038/s41598-022-09545-7)
Supplement: Supplementary file 1 — Supplementary Table 1. [file 41598_2022_9545_MOESM1_ESM.docx]

Supplementary table 1. Embryo quality score of two transferred embryos

Cleavage stage embryos with at least 6 blastomeres and less than 40% fragmentation were defined as viable embryos. Among them, those with at least 7 even and homogeneous blastomeres and less than 20% fragmentation were considered as top quality embryos (Top) (Van Royen et al., 2001), and the other viable embryos were marked as fair good embryos (Fair).

Blastocyst stage embryos were assessed according to Gardner’s criteria (Gardner et al., 2000). Inner cell mass/trophectoderm scored AA, AB, BA, BB were considered as top quality blastocysts (Top), and AC, BC, CA, CB as fair good embryos (Fair). Blastocysts scored CC were excluded in this study.

| Embryo Quality Score | Cleavage stage | Blastocyst stage |
| --- | --- | --- |
| 1 | Top + Top | Top + Top |
| 2 | Top + Fair | Top + Fair |
| 3 | Fair + Fair | Fair + Fair |
